# Assessing the Effectiveness of Engaging Patients and Their Families in the Three-Step Fall Prevention Process Across Modalities of an Evidence-Based Fall Prevention Toolkit: An Implementation Science Study

**DOI:** 10.2196/10008

**Published:** 2019-01-21

**Authors:** Megan Duckworth, Jason Adelman, Katherine Belategui, Zinnia Feliciano, Emily Jackson, Srijesa Khasnabish, I-Fong Sun Lehman, Mary Ellen Lindros, Heather Mortimer, Kasey Ryan, Maureen Scanlan, Linda Berger Spivack, Shao Ping Yu, David Westfall Bates, Patricia C Dykes

**Affiliations:** 1 Division of General Internal Medicine Brigham and Women's Hospital Boston, MA United States; 2 NewYork-Presbyterian Hospital Columbia University Manhattan, NY United States; 3 Montefiore Medical Center Bronx, NY United States; 4 Harvard Medical School Boston, MA United States

**Keywords:** clinical decision support, fall prevention, fall prevention toolkit, health information technology, implementation science, patient safety

## Abstract

**Background:**

Patient falls are a major problem in hospitals. The development of a Patient-Centered Fall Prevention Toolkit, Fall TIPS (Tailoring Interventions for Patient Safety), reduced falls by 25% in acute care hospitals by leveraging health information technology to complete the 3-step fall prevention process—(1) conduct fall risk assessments; (2) develop tailored fall prevention plans with the evidence-based interventions; and (3) consistently implement the plan. We learned that Fall TIPS was most effective when patients and family were engaged in all 3 steps of the fall prevention process. Over the past decade, our team developed 3 Fall TIPS modalities—the original electronic health record (EHR) version, a laminated paper version that uses color to provide clinical decision support linking patient-specific risk factors to the interventions, and a bedside display version that automatically populates the bedside monitor with the patients’ fall prevention plan based on the clinical documentation in the EHR. However, the relative effectiveness of each Fall TIPS modality for engaging patients and family in the 3-step fall prevention process remains unknown.

**Objective:**

This study aims to examine if the Fall TIPS modality impacts patient engagement in the 3-step fall prevention process and thus Fall TIPS efficacy.

**Methods:**

To assess patient engagement in the 3-step fall prevention process, we conducted random audits with the question, “Does the patient/family member know their fall prevention plan?” In addition, audits were conducted to measure adherence, defined by the presence of the Fall TIPS poster at the bedside. Champions from 3 hospitals reported data from April to June 2017 on 6 neurology and 7 medical units. Peer-to-peer feedback to reiterate the best practice for patient engagement was central to data collection.

**Results:**

Overall, 1209 audits were submitted for the patient engagement measure and 1401 for the presence of the Fall TIPS poster at the bedside. All units reached 80% adherence for both measures. While some units maintained high levels of patient engagement and adherence with the poster protocol, others showed improvement over time, reaching clinically significant adherence (>80%) by the final month of data collection.

**Conclusions:**

Each Fall TIPS modality effectively facilitates patient engagement in the 3-step fall prevention process, suggesting all 3 can be used to integrate evidence-based fall prevention practices into the clinical workflow. The 3 Fall TIPS modalities may prove an effective strategy for the spread, allowing diverse institutions to choose the modality that fits with the organizational culture and health information technology infrastructure.

## Introduction

Falls are a public health problem. Hospitalization increases the risk of falls [1]. In US hospitals, fall rates range from 3.3 to 11.5 falls per 1000 patient days, with about 25% of in-hospital falls resulting in injury [2]. Falls lead to longer lengths of stay, increased costs, and can have severe psychological impacts on patients [3-5].

Fall prevention research has defined the risks that contribute to in-hospital falls and established valid and reliable fall risk assessment tools [6-8]. However, there was a dearth of research regarding fall prevention protocols that link fall risk factors to evidence-based interventions. After identifying this gap, our team interviewed patients who had fallen and their care team members to determine perceptions of why hospitalized patients fall and interventions that could be effective and feasible in the hospital setting [9,10]. The results led to the conclusion that preventing falls in the hospital is a 3-step process as follows: (1) conducting fall risk assessments; (2) developing a tailored fall prevention plan; and (3) implementing that plan consistently along with universal fall precautions. These qualitative results led to the development of the electronic Fall TIPS (Tailoring Interventions for Patient Safety) Toolkit, an intervention that leverages health information technology to provide clinical decision support linking the fall risk assessment to tailored interventions. The Fall TIPS Toolkit was tested in a randomized control trial on >10,000 patients and showed a 25% reduction in fall rates [11]. The results of this study established the linkage between conducting fall risk assessments and implementing tailored, evidence-based interventions to prevent in-hospital falls.

We conducted a case–control study to understand why patients who received the Fall TIPS intervention fell. The most common reason was that patients did not follow their fall prevention plan [12]. Patients often do not believe they are at risk for falls while hospitalized [10]. These data support the hypothesis that simply teaching patients after completing the fall risk assessment and plan is insufficient; patients must be engaged throughout all 3 steps of the fall prevention process. This engagement protocol can improve the partnership with patients for implementing the plan, which is key in further reducing fall with injury rates in hospitals [13].

Despite the widespread adoption of electronic health records (EHRs) in recent years [14], not every health system can adopt the electronic Fall TIPS Toolkit. Barriers include unsophisticated EHR platforms, lack of funds for the toolkit build, and lack of staff engagement to successfully support the roll out. To address the barriers and facilitate spread, our team developed, tested, and iteratively refined a laminated version of the Fall TIPS Toolkit in collaboration with health systems engineers [14,15]. The laminated version of the Fall TIPS Toolkit preserves the clinical decision support of the electronic version by integrating color to provide the linkage between patients’ fall risk factors and the evidence-based interventions. It is a low-tech, patient-friendly solution with few barriers to adoption [16]. It is available in both English and Spanish to serve a diverse patient population.

The laminated Fall TIPS Toolkit was evaluated in a 6-month pilot at 2 sites. In a study, adherence to use of the tool was high (>80%), and patient fall with injury rates declined at both sites [13]. This pilot demonstrated the efficacy of the laminated version of the Fall TIPS Toolkit when it is integrated into the workflow and indicates that at least 80% adherence to the Fall TIPS protocol is clinically significant for lowering fall-related injury rates [13].

To further improve the flexibility, adoption, and patient-centeredness of the Fall TIPS Toolkit, we have also developed a patient safety bedside display. This automatically displays each patient’s fall prevention plan on the bedside monitor once a nurse has documented the risk assessment and tailored the fall prevention plan in the EHR. This level of automation provides a guarantee that the information displayed at the bedside is up-to-date and is a means of displaying the fall prevention plan in rooms that do not have a visible location to hang the Fall TIPS poster [17].

These 3 modalities, the electronic Fall TIPS Toolkit, the laminated Fall TIPS Toolkit, and the patient safety bedside display, were developed to engage patients in the 3-step fall prevention process and to spread and integrate evidence into practice regardless of an institution’s technical capabilities and local factors. The purpose of this study is to assess the effectiveness for engaging patients and family in the 3-step fall prevention process (as defined by patient/family knowledge of their personalized fall risk factors and prevention plan) of each of the Fall TIPS modalities.

## Methods

We conducted this study at Brigham and Women’s Hospital (Boston, MA, USA), Montefiore Medical Center (MMC; Bronx, NY, USA), and NewYork-Presbyterian Hospital (Manhattan, NY, USA). Each site incorporated the Fall TIPS fall prevention process into practice and built the clinical decision support provided by Fall TIPS into the EHR. At each site, nurses complete the Fall TIPS risk assessment and tailored plan and then documented it in the EHR. Furthermore, the 3 modalities were utilized to present and communicate the patients’ fall risk factors and tailored fall prevention plan.

**Figure 1:**
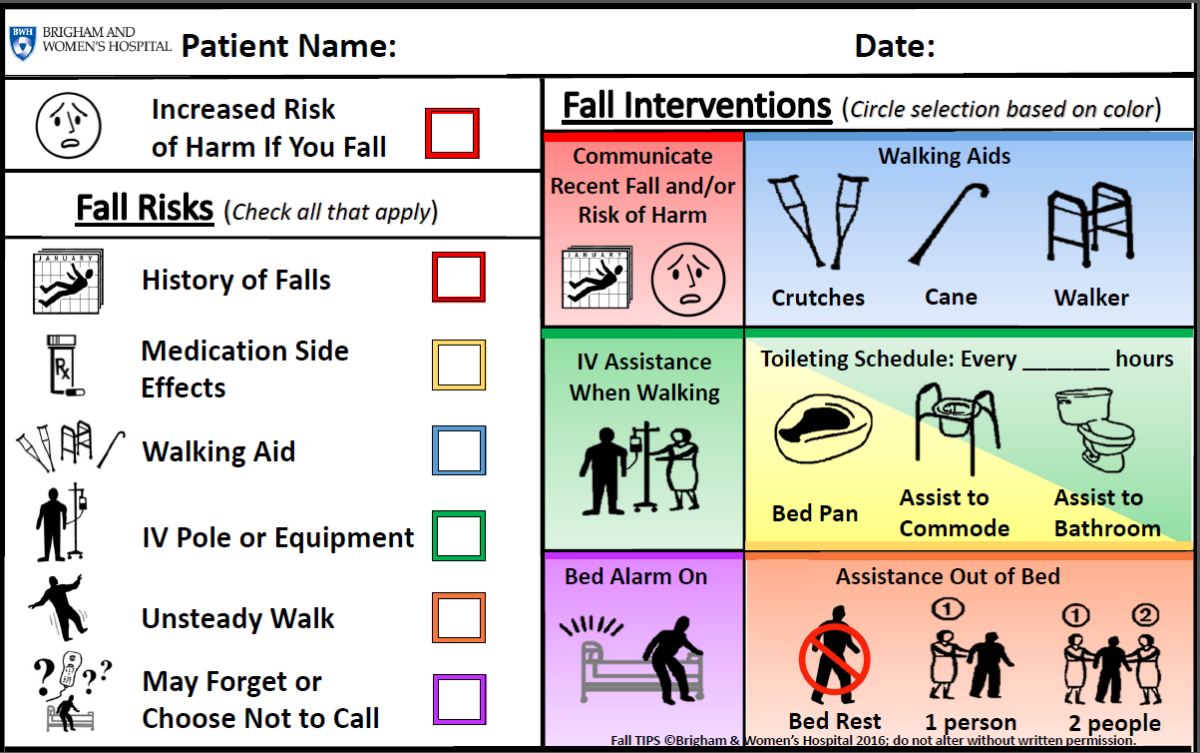
Modality 1: Laminated Paper Fall TIPS bedside poster, on which a nurse manually documents a patient’s fall risks and tailored intervention plan.

**Figure 2:**
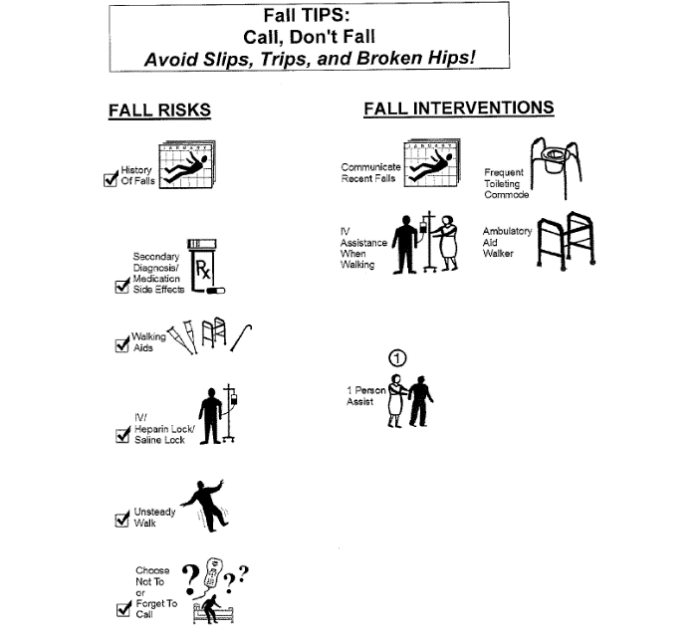
Modality 2: The Electronic Fall TIPS bedside poster, generated by nurse documentation of the personalized fall prevention plan in the electronic health record.

**Figure 3:**
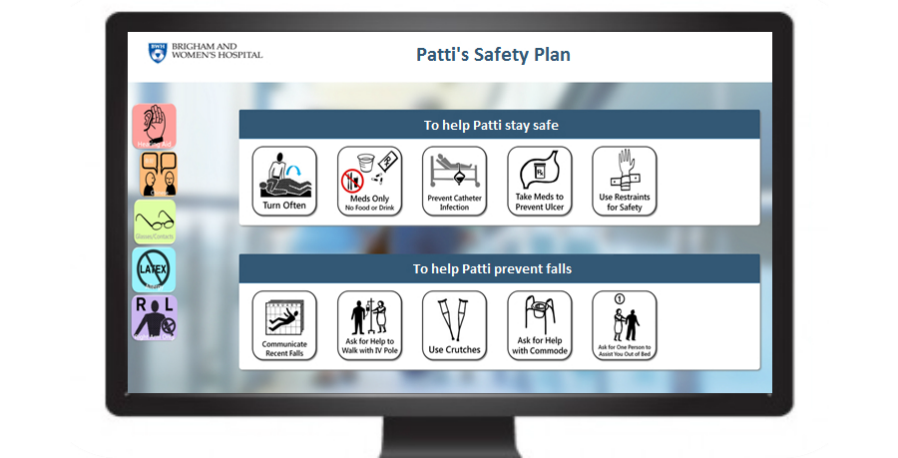
Modality 3: Patient safety bedside display of the Fall TIPS personalized fall prevention plan which is automatically displayed as the screensaver on the computer monitor in the patient’s room after nurse documentation in the electronic health record.

The 3 bedside modalities are as follows: (1) the laminated Fall TIPS poster (Figure 1); (2) electronic Fall TIPS poster (Figure 2); and the paperless patient safety bedside display (Figure 3).

To assess the effectiveness of engaging patients in the 3-step fall prevention process across the Fall TIPS modalities, random audits were conducted asking, “Does the patient/family member know their fall prevention plan?” In addition, random audits measured protocol adherence, defined as the presence of the Fall TIPS fall prevention plan at the bedside.

Weekly data were reported April-June 2017. Fall prevention nurse champions randomly selected, at previously unannounced times, eligible patients or family members for the audit. The inclusion criteria were as follows: patients must be aged ≥18 years; either alert and oriented or have family present and involved in care; English or Spanish speaking; and have a length of stay >24 hours (to allow nurse time to engage patient and family). To conduct the audit, nurse champions verbally asked patients or family members about their knowledge and engagement with their fall prevention plan.

Data were collected on 6 Neurology units and 7 medical or medical-surgical units; these units were chosen for the sample because the team sought to include an analysis of Modality 3 in the study. Modality 3 had only been deployed on 3 Neurology and 2 medical or medical-surgical units at Brigham and Women’s Hospital as part of an Agency for Healthcare Research and Quality-funded Patient Safety Learning Lab grant. Neurology and medical or medical-surgical units were then selected for inclusion at MMC and NewYork-Presbyterian Hospital to have equal representation of these services at each hospital. Multimedia Appendix 1 presents a table of the Fall TIPS modality utilized at each hospital by the unit.

The main outcomes measured were the percentage of patients and family members who reported knowing their personal fall risk factors and plan across the 3 Fall TIPS modalities and protocol adherence measured as the display of the personalized fall prevention plan at the bedside.

## Results

**Figure 4:**
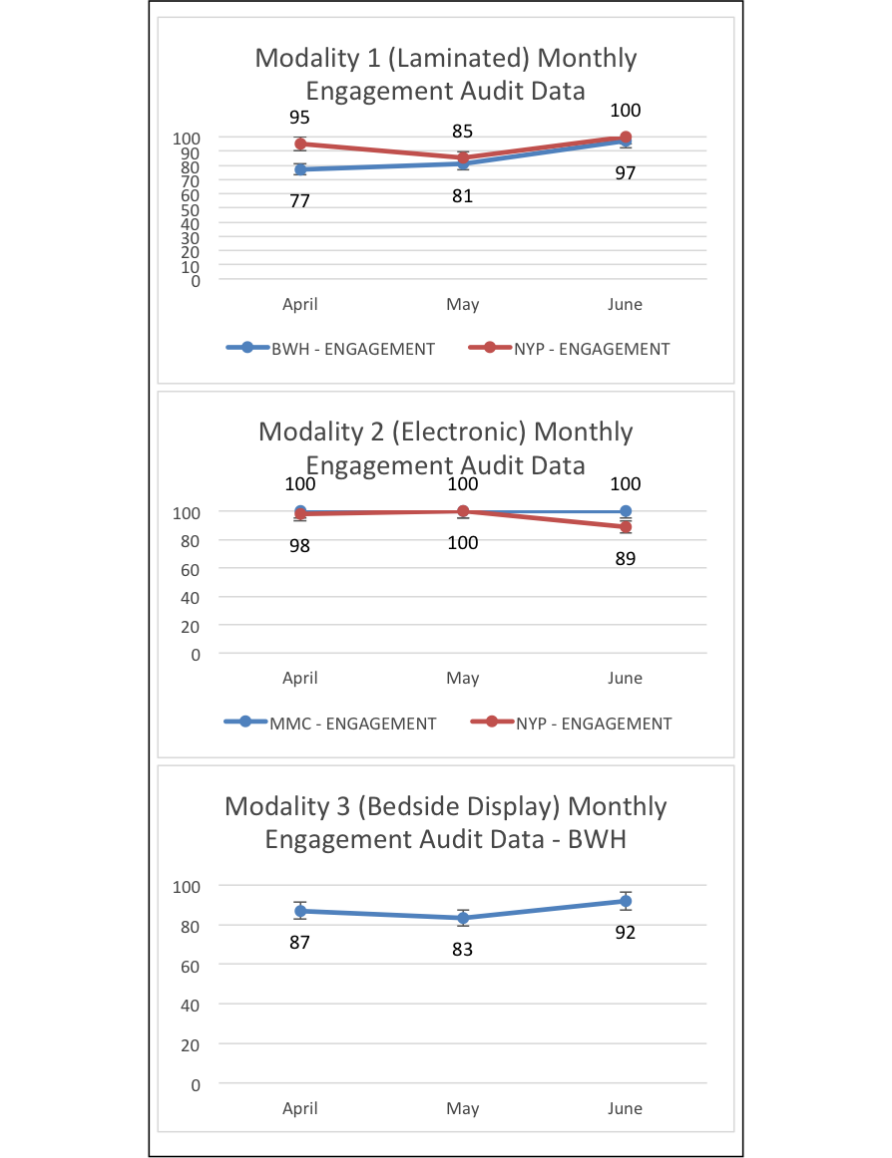
Patient engagement audit data by modality from April to June 2017. BWH: Brigham and Women’s Hospital. NYP: NewYork-Presbyterian Hospital; MMC: Montefiore Medical Center.

**Figure 5:**
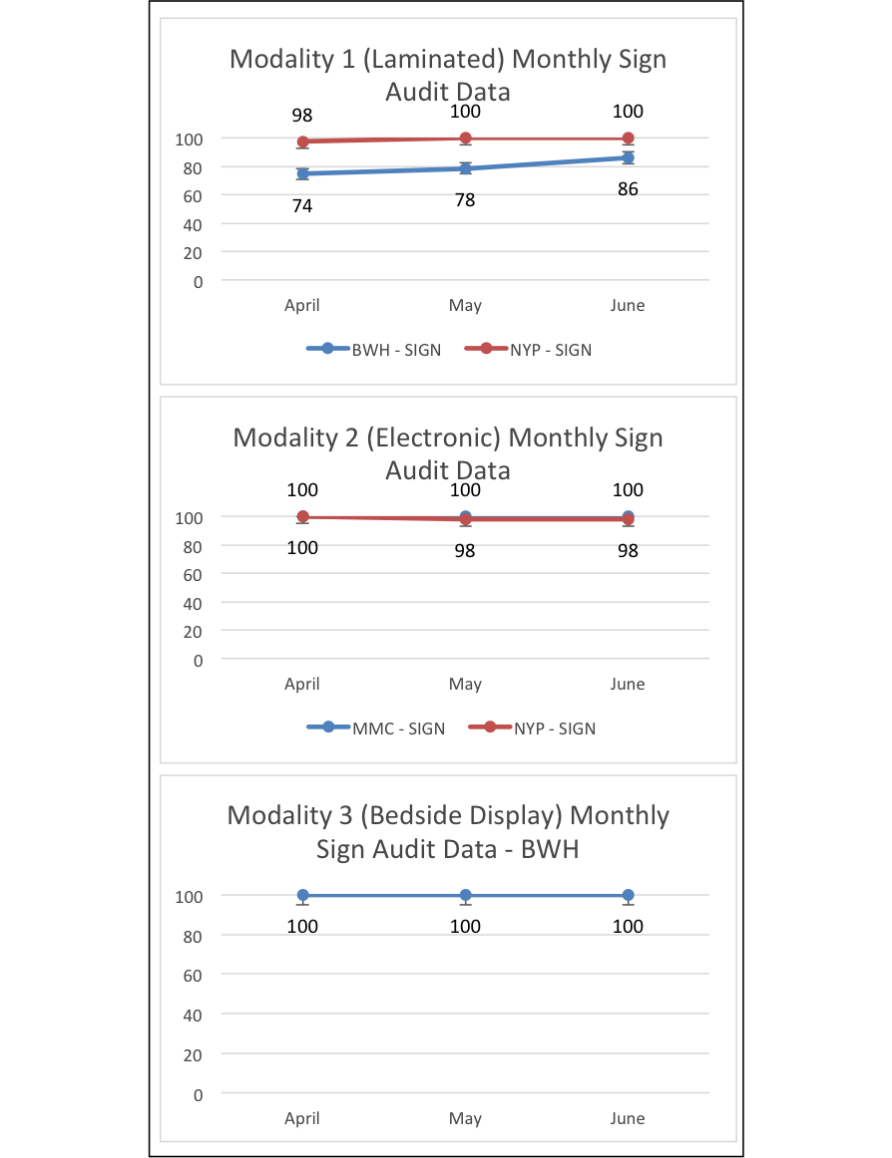
Adherence to the Fall TIPS protocol, as measured by the presence of the personalized fall prevention plan at the bedside, by modality from April to June 2017. BWH: Brigham and Women’s Hospital; NYP: NewYork-Presbyterian Hospital; MMC: Montefiore Medical Center.

Nurses submitted 1209 audits for the patient engagement measure and 1401 for the presence of the Fall TIPS poster at the bedside. The sample included a diverse population of patients; at MMC, 37.78% (481/1273) of patients reported Hispanic ethnicity, where the Spanish tool is frequently utilized. Table 1 presents the patients’ demographics. The average ages of patients at the Brigham and Women’s Hospital, MMC, and NewYork-Presbyterian Hospital were 60.5, 60.1, and 60.3 years, respectively. All units reached at least 80% adherence for both measures by the last month of data collection. Figure 4 provides patient engagement audit data over time. Figure 5 provides adherence data to the Fall TIPS protocol as measured by the presence of the personalized fall prevention plan at the bedside. Multimedia Appendices 2 and 3 present audit data counts over time at each site.

**Table 1:** Patients’ characteristics by the site.

Characteristics		Brigham and Women’s Hospital (n=1842), n (%)	Montefiore Medical Center (n=1273), n (%)	NewYork-Presbyterian Hospital (n=2582), n (%)
Female		999 (54.23)	709 (55.69)	1343 (52.01)
**Race**				
	American Indian or Native Alaskan	4 (0.22)	3 (0.24)	5 (0.19)
	Asian	43 (2.33)	21 (1.65)	76 (2.94)
	Black or African American	259 (14.06)	416 (32.68)	310 (12.01)
	Declined	13 (0.71)	110 (8.64)	34 (1.32)
	Native Hawaiian or Other Pacific Islander	2 (0.11)	1 (0.08)	10 (0.39)
	Other	170 (9.23)	509 (39.98)	23 (0.89)
	Unavailable	32 (1.74)	25 (1.96)	1433 (55.50)
	White or Caucasian	1319 (71.61)	188 (14.77)	691 (26.76)
**Ethnicity**				
	Declined	2 (0.11)	107 (8.41)	33 (1.28)
	Hispanic	172 (9.34)	481 (37.78)	361 (13.98)
	Non-Hispanic	1593 (86.48)	615 (48.31)	557 (21.57)
	Unavailable	75 (4.07)	70 (5.49)	1631 (63.17)

## Discussion

### Principal Findings

This study sought to determine if there was variability in the ability to engage patients in the 3-step fall prevention process across the Fall TIPS modalities. The results illustrate little difference in the ability to engage patients across the 3 modalities. All units, regardless of the modality and site, reached clinically significant rates (>80%) of patient engagement and adherence with the sign protocol to reduce fall and fall with injury rates [13]. This suggests that each Fall TIPS modality is effective at engaging patients in the 3-step fall prevention process and so can be used to implement the evidence into practice.

The different levels of automation provide flexibility for institutions to individualize their Fall TIPS implementation approach. Institutions must assess which modality is most appropriate, considering factors like EHR capability, the commitment of health information technology support, financial constraints and local geographic realities, such as room layouts, or whether there are uniform bedside monitors. Further research investigating how each Fall TIPS modality impacts fall and fall with injury rates is needed. In practice, Fall TIPS is used in >100 hospitals and continues to spread. Interested hospitals have free access to the Fall TIPS Fall Prevention Toolkit and training materials through the Fall TIPS Collaborative.

### Limitations

As Fall TIPS was implemented at 3 different institutions, differences in the communication channels, social systems, the support from leadership and the timing of Fall TIPS implementation pose limitations to this study. Some of these factors are discussed elsewhere [13]. Each of these factors could have confounded the levels of patient engagement with fall prevention as related to the Fall TIPS modality utilized and protocol adherence.

Finally, this is an implementation science study. The study was not designed to randomize units to each modality but to assess the efficacy of the Fall TIPS modalities within the existing institutional frameworks. This was a study of the uptake of evidence-based practice across modalities. The implementation of practice into the workflow does not allow for perfect comparability but demonstrates that achieving clinically significant rates of adherence to an evidence-based fall prevention program in the workflow is possible.

### Conclusions

Fall TIPS is an evidence-based fall prevention intervention that provides built-in clinical decision support to engage patients and family in the 3-step fall prevention process. It has been iteratively developed and refined to include 3 modalities with varying degrees of automation while preserving the clinical decision support inherent to Fall TIPS. The 3 modalities provide flexibility for health systems with different capabilities to integrate the fall prevention evidence into practice.

This study demonstrates that across the 3 modalities, the laminated Fall TIPS Toolkit, the electronic Fall TIPS Toolkit, and the patient safety bedside display of Fall TIPS, there is no significant clinical difference in ability to engage patients. Previous research has shown that patient engagement is the crux of improving fall rates and that at least 80% adherence with the Fall TIPS protocol is clinically significant for doing so [13]. Therefore, the ability to engage patients corresponds with the efficacy of the Fall TIPS modalities. Overall, this study suggests that providing 3 Fall TIPS modalities is an effective and flexible approach for promoting adoption and spread.

## Appendix

Multimedia Appendix 1 Fall TIPS modality utilized at each hospital by unit.

Multimedia Appendix 2 Monthly engagement audit data by modality at each site.

Multimedia Appendix 3 Monthly protocol adherence audit data by modality at each site.

## Abbreviations

EHR: electronic health record
MMC: Montefiore Medical Center
TIPS: Tailoring Interventions for Patient Safety
