# Supplementary figures and images for: Assessing the Effectiveness of Engaging Patients and Their Families in the Three-Step Fall Prevention Process Across Modalities of an Evidence-Based Fall Prevention Toolkit: An Implementation Science Study
Source: J Med Internet Res. 2019 Jan 21;21(1):e10008. doi: 10.2196/10008 (PMC6360379; doi:10.2196/10008)

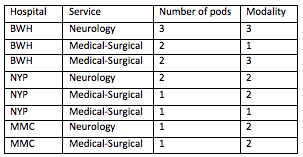

Supplement: Multimedia Appendix 1 [file jmir_v21i1e10008_app1.png]

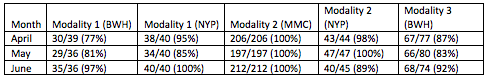

Supplement: Multimedia Appendix 2 [file jmir_v21i1e10008_app2.png]

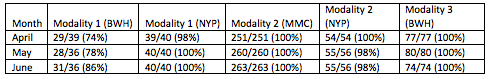

Supplement: Multimedia Appendix 3 [file jmir_v21i1e10008_app3.png]
